# Preventing long term relapsing tinea unguium with topical anti-fungal cream: a case report

**DOI:** 10.1186/1757-1626-2-70

**Published:** 2009-01-21

**Authors:** Bruce Arroll, Amanda Oakley

**Affiliations:** 1 University of Auckland, Private Bag 92019, Auckland, New Zealand; 2 Department of Dermatology, Waikato Hospital, Private Bag 3200, Hamilton 3001, New Zealand

## Abstract

**Background:**

The patient was aged 34 when he consulted a dermatologist in Vancouver BC with onychomycosis affecting the right great toenail.

**Case presentation:**

*Trichophyton rubrum *was cultured from nail clippings. Griseofulvin was taken for 6 months, resulting in clinical and mycological cure. Over the next 27 years there were multiple relapses. Each course of treatment with oral terbinafine (for up to 18 months) or itraconazole resulted in clinical and mycological cure. A dermatological colleague suggested the reason for relapse was likely to be self re-infection.

**Conclusion:**

No clinical relapse has occurred with once-weekly miconazole cream applied to the toenail and webspaces of the right foot over the last four years.

## Background

Onychomycosis is fungal infection affecting fingernails or toenails, and may be due to dermatophyte, yeast or mould. Tinea unguium is the term used when a dermatophyte has been identified on culture.

Oral medication is recommended for extensive or symptomatic tinea unguium, and is particularly appropriate in diabetics at risk of secondary bacterial infection. The most effect fungicide is terbinafine with reported 70% cure rates for toenails and 80% for fingernails. Itraconazole also has a high success rate. Recurrence is common.[1]

Oral antifungal medications may be taken intermittently or continuously, and typically a course of 3 to 6 months is prescribed, depending on response and severity. Prolonged courses may be prescribed in older patients but treatment may fail because of slow-growing nails and poor peripheral circulation. Minor side effects are common. Terbinafine occasionally results in serious adverse reactions including toxic epidermal necrolysis, hepatitis and agranulocytosis. Drug interactions, cardiac and hepatic toxicity are the main concern with azoles.[2]

Topical antifungal agents are often prescribed for tinea unguium. They have impressive in vitro cure rates. However they are rarely curative in vivo [3], presumably because of poor penetration into the nail plate, frequent involvement of inaccessible proximal nail matrix, and non-adherence to instructions. It is understandable that patients would get tired of applying a solution or lacquer once or twice weekly for a year or longer, if no improvement is seen within a few weeks of use. Therefore topical medications should be restricted to confirmed distal infection of one or two nails only, in those able to debride their own nails. In more extensive disease they may be used concurrently with oral drugs, or alone if oral medications are contraindicated to reduce spread to cutaneous sites.

A Medline search of tinea unguium and recurrence revealed few references and little evidence regarding the use of topical medication in preventing relapse.[4]

## Case presentation

Age 35 at time of diagnosis 56 at time of writing

Sex Male

Occupation Doctor

Ethnicity Caucasian

Weight 90 kg

Height 1.87 m

Social. The patient was working in Vancouver BC Canada. He now lives in New Zealand

Medical history (At the time of diagnosis, hand eczema and recurrent tinea pedis; subsequently hypertension (1994), currently treated with chlorthalidone, lisinopril and metoprolol.)

Family history (Mother died of a myelodysplasia, aged 86 years. She also had recurrent melanoma on one leg. Father died at age 87 of congestive heart failure and multiple myeloma. Sister has asthma and hypertension.

Non smoker

Alcohol less than 1 standard drink per week

Current medications: Chlorthalidone 25 mg daily, lisinopril 20 mg daily and metoprolol succinate 190 mg per day.

## The description of the case

**Figure 1:**
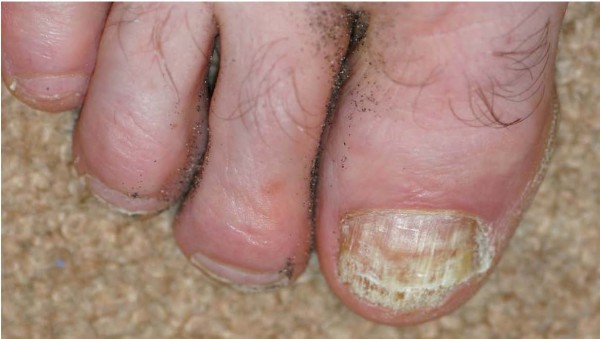
**Photo 1 during a relapse 1994**.

Symptoms presented with: There were no symptoms. The main concerns were the appearance, and risk of transmitting infection to family members. Signs on presentation: Distal and lateral aspects of the right toenail were yellow, thickened and opacified, with onycholysis and irregular subungual hyperkeratosis (figure 1). All the other nails were normal. There was also tinea pedis in the form of toeweb maceration in both 4^th ^to 5^th ^webspaces on the toes.

Diagnostic tests. Mycological testing of the nail revealed fungal elements on microscopy and *Trichophyton rubrum *on culture.

Any imaging carried out; None

Primary diagnosis: Tinea pedis and tinea unguium due to *T rubrum*

Any subsequent diagnoses. Recurrent tinea pedis and tinea unguium

Pharmacological treatments – The first treatment was griseofulvin 1000 mg per day and was taken for 6 months. Itraconazole was later used in a dose of 400 mg daily for one week, repeated each month for 6 months. Two courses of pulse Itraconazole and continuous therapy with 400 mg daily for 6 months followed. Multiple courses of terbinafine 250 mg per day for 6 months were tried and one course went for 18 months.

Surgical interventions. Nil

Any other interventions: At the age of 52 he started applying topical miconazole once per week to the affected large toe and to the toe clefts of his right foot.

**Figure 2:**
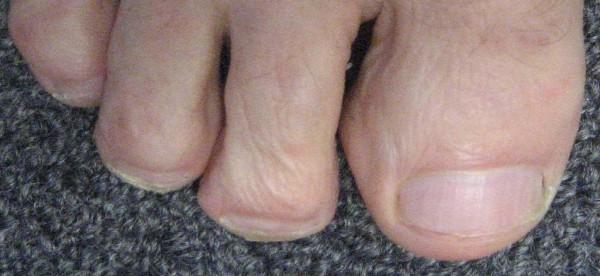
**Photo 2 current condition of toe 2008**.

Outcome of the case: All courses of oral antifungal medications resulted in a clinical cure. Mycological cure was confirmed on three occasions. Until recently, apparent cure was always followed by relapse. However, there has been no recurrence of tinea unguium since starting prophylactic topical miconazole (figure 2).

## Discussion

The patient has been extremely relieved by the outcome of topical prophylaxis, as he has been concerned about potential harm of repeated courses of oral antifungal agents.

The relapse rate for onychomycosis is known to be high and increases with time: "The relapse rate increased from 8. 3% at month 12 to 19.4% at month 24 and to 22.2% at month 36." [5].

Risk factors for recurrent onychomycosis are older age, abnormal nail morphology (especially a thickened nail plate due to trauma and psoriasis), immunodeficiency and genetic factors. A search of medical literature and textbooks suggests "Successful treatment does not prevent future reinfection", but specific advice on how to prevent re-infection is rarely described.[3] Topical ciclopirox or amorolfine lacquer for the nail is often suggested. Preventative measures include protective footwear and cotton socks, antifungal or absorbent powders, and frequent nail clipping. Old shoes often harbour large numbers of infectious organisms and should be discarded or treated with disinfectants or antifungal powders.[6]

It is recognised that tinea pedis needs to be treated vigorously, yet a double-blind trial reported no statistical difference between miconazole powder and placebo used to prevent recurrence of onychomycosis in 48 patients. The small sample size indicates type 2 statistical error was possible (i.e. no change found when a true difference exists). [7]

In vivo, miconazole is effctive for tinea pedis but it is not effective for active onychomycosis. Topical miconazole is active in vitro against *T rubrum*, although less potent than terbinafine and itraconazole. An increase in minimum inhibitory concentration has been noted in organisms isolated post treatment compared to pretreatment of onychomycosis. [8] We were unable to find any reports of clinical resistance to topical miconazole, but this is a theoretical concern with prophylactic use of this drug.

Our patient has been lucky and has finally achieved longterm remission; we think this is likely due to topical miconazole prophylaxis, but this impossible to prove.

## Conclusion

We have described a case in which sustained remission from onychomycosis was achieved with weekly miconazole cream applied to the affected big toe and toe clefts. Miconazole may reduce the need for further potentially hazardous oral treatment in individuals with recurrent onychomycosis. However, it is important to first achieve clinical and mycological remission with oral therapy.

## Consent

Written informed consent was obtained from the patient for publication of this case report and accompanying images. A copy of the written consent is available for review by the Editor-in-Chief of this journal.

## Competing interests

BA is on the advisory board for the educational seminars run by PHARMAC, New Zealand's government funded drug purchasing agency. He is also on the primary care committee of the Future Forum and educational foundation funded by Astra Zeneca (UK). He has accepted travel and conference funding from Sanofi Aventis.

AO has received fees from PHARMAC for educational seminars. She is the Website Manager of the New Zealand Dermatological Society, NZ DermNet, which is funded mainly by pharmaceutical companies, including those that market antifungal agents.

## Authors' contributions

BA provided the photographs and the idea for the paper. AO provided the description of the toes and added many pieces of relevant clinical information and references. Both edited the paper.
